# The Crucial Role of Diet Therapy and Selenium on the Evolution of Clinical and Paraclinical Parameters in Patients with Metabolic Syndrome

**DOI:** 10.1155/2023/6632197

**Published:** 2023-09-25

**Authors:** Adrian Marius Danciu, Timea Claudia Ghitea, Alexa Florina Bungau, Cosmin Mihai Vesa

**Affiliations:** ^1^Doctoral School of Biological and Biomedical Sciences, University of Oradea, 410087 Oradea, Romania; ^2^Faculty of Medicine and Pharmacy, Pharmacy Department, University of Oradea, 410068 Oradea, Romania; ^3^Faculty of Medicine and Pharmacy, Medicine Department, University of Oradea, 410068 Oradea, Romania

## Abstract

Oxidative stress (OS) is associated with metabolic syndrome (MS) and represents a complex disease association that has become a major challenge in the field of public health. The aim of this study was to investigate the effectiveness of introducing selenium in the management of OS, while considering a balanced diet based on a healthy lifestyle and dietary therapy. A total of 206 individuals participated voluntarily in the study, divided into three groups: the control group with 35 individuals (17.0%) designated as control lot (LC), the group undergoing diet therapy with 119 individuals (57.8%) designated as diet therapy lot (LD), and the group undergoing diet therapy supplemented with selenium consisting of 52 individuals (25.2%) designated as diet therapy with selenium lot (LD + Se). The study assessed various clinical parameters (such as body mass index (BMI), body weight status, fat mass, visceral fat, and sarcopenic index), paraclinical parameters (including HOMA index, cholesterol, triglycerides, C-reactive protein, and glycosylated haemoglobin (HGS)), as well as OS parameters (measured using the FORD test, FORT test, and MIXED test). The LD + Se group demonstrated the most favourable results in terms of BMI reduction, decreased fat and visceral mass, reduced levels of C-reactive protein, and improved glycosylated haemoglobin levels. By implementing a balanced diet therapy and supplementing the diet with selenium, it was possible to achieve a reduction in adipose tissue and glycosylated haemoglobin levels, ultimately contributing to the reduction of OS in the body.

## 1. Introduction

Oxidative stress (OS) and soluble factors are associated with a sedentary lifestyle, abdominal obesity, and metabolic pathways which are considered the main molecular and cellular mechanisms involved in the imbalance observed in metabolic syndrome (MS). These findings offer new opportunities for prevention and treatment strategies [1]. Recent research has been devoted to examining the effects of metabolic imbalance on individual organs, extending beyond the pathologies typically associated with MS. Metabolism, responsible for converting nutrients into the energy required for sustaining life, is disrupted in MS, not limited to abdominal obesity alone [2].

**Figure 1:**
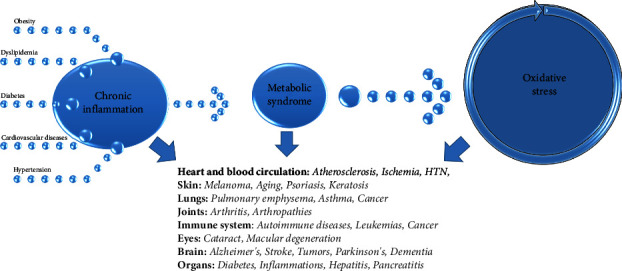
The effect of oxidative stress on the body [6, 7].

OS, caused by elevated levels of reactive oxygen species (ROS), is linked to chronic low-grade inflammation and exerts detrimental effects on human health. Obesity, particularly excess abdominal fat mass, plays a pivotal role in the development of cardiovascular disease through the abnormal production of adipokines and insulin resistance [3]. MS is primarily analyzed from an endocrinological perspective and it is characterized as a combination of clinical and metabolic factors, such as insulin resistance, hyperglycemia, hypertension, dyslipidemia, and abdominal obesity [4, 5], presented in Figure 1.

A current research focused on the disruption of metabolic balance and has identified myokines and *β*-aminoisobutyric acid as important factors in the prevention and treatment of MS [1]. OS plays a fundamental role in the development and progression of MS, and it has been observed that MS patients exhibit high levels of ROS and decreased antioxidant defenses. These findings highlight the significance of maintaining a balance between oxidants and antioxidants [8] to address the oxidant-antioxidant imbalance in MS [9]. Liver diseases, including steatosis and nonalcoholic fatty liver disease (NAFLD) [10, 11], are closely linked to MS. A more precise definition and improved understanding of MS could be beneficial in preventing the development of these liver diseases [4]. The liver plays a crucial role in regulating iron balance, and this process can be compromised in diseases associated with MS. Research has demonstrated a connection between iron-induced liver injury and elevated levels of ROS, which are implicated in the pathogenesis of MS. Hence, it is imperative to intervene and manage the effects of MS on liver function due to the substantial impact of liver disease on overall health and its global significance in maintaining optimal liver function [12].

Over time, research on MS has persistently delved into the intricate mechanisms that underlie this syndrome. Gaining a comprehensive understanding of these cellular-level mechanisms presents us with the prospect of developing personalized interventions and therapies. Moreover, it enables us to comprehend the associations between MS and other systemic conditions, and in some cases, even ascertain its role as a causative factor [13].

Mitochondria play a crucial role in adenosine triphosphate (ATP) production while also serving as a significant source of ROS. The process of electron transfer in the electron transport chain can generate superoxide and hydrogen peroxide radicals. These radicals have the potential to cause cellular damage, including harm to membranes, proteins, enzymes, and deoxyribonucleic acid (DNA), which may ultimately result in cell death. Nonetheless, mitochondria possess an effective antioxidant mechanism that minimizes the production of hazardous hydroxyl radicals. When mitochondrial function is compromised, excessive ROS production can occur, leading to OS and cellular damage. This OS is recognized as a pivotal factor in the pathogenesis of various diseases, including MS. Additionally, ROS can act as intracellular messengers, influencing cellular signaling and metabolism. Increased ROS production may contribute to the development of MS by augmenting OS and initiating metabolic reprogramming [14, 15].

Due to the alterations in lipid composition mediated by ROS, other products derived from oxidation, such as oxidative low-density lipoprotein (Ox-LDL), are believed to contribute significantly to cardiovascular disease. Ox-LDL refers to particles derived from circulating LDL that may contain peroxides or their degradation products formed within the LDL molecule or elsewhere in the body. Different mechanisms have been observed to regulate lipoprotein lipase (LPL) expression and fatty acid accumulation in adipocytes [16–18]. There are several possible explanations for this association [19]. First, a high glycemic load diet results in an increase in circulating levels of insulin-like growth factor 1 (IGF-1) and insulin [20–22]. This process further contributes to the development of metabolic diseases [23]. Increased milk consumption has been associated with the potential effect of triggering heightened production of insulin-like growth factor 1 (IGF-1) by the liver. Furthermore, this elevated consumption of milk may also result in an augmentation of insulin levels circulating within the bloodstream. Both IGF-1 and insulin are not completely inactivated by processes such as pasteurization, homogenization, and digestion. While the Mediterranean diet advises moderate daily dairy consumption [24], an excessive intake of dairy can potentially yield effects akin to consuming a meal with a high glycemic load [25–27].

The studies revealed that patients with diabetes exhibited increased FORT levels (2.86 ± 0.56 mmol/L H_2_O_2_) compared to the control group (1.87 ± 0.26 mmol/L H_2_O_2_) (*P* < 0.0001). Additionally, FORD levels were lower in diabetic patients (1.23 ± 0.18 mmol/L Trolox) than those in the control group (1.34 ± 0.14 mmol/L Trolox) (*P* < 0.01). The intra-assay and interassay coefficients of variation were determined to be 3.7% and 6.2%, respectively, for FORT, and 4.2% and 6.6%, respectively, for FORD. These findings suggest that the measurement of oxygen free radicals and defense mechanisms against them play a significant role in understanding and assessing OS generation [28].

The pathogenesis of MS encompasses various factors, and multiple studies provide evidence supporting the involvement of OS and chronic inflammation in the development of metabolic diseases. OS is characterized by an imbalance between the oxidative and antioxidant systems within cells and tissues, resulting in the excessive generation of oxidative free radicals and ROS. Elevated levels of ROS can inflict damage upon cellular proteins, lipids, and nucleic acids, thereby leading to cellular dysfunction. This disruption can affect energy metabolism, alter cell signaling and cell cycle regulation, induce genetic mutations, impact cellular transport mechanisms, and overall diminish biological activity. Additionally, excessive ROS production can activate the immune system and trigger inflammation, contributing to the pathogenesis of MS [29].

Based on studies in PubMed, we utilized a period and dose of selenium (30 days and 200 mcg) for which no risks were reported. Selenium is involved in the functioning of over 30 selenoproteins, where it is incorporated as a selenocysteine in their active sites. Key selenoproteins include glutathione peroxidase, thioredoxin reductase, and iodothyronine deiodinase, all of which are essential for maintaining proper thyroid function and act as antioxidant enzymes. Approximately 60% of serum selenium is associated with selenoprotein P, which acts as a transport protein for selenium and contains 10 selenium atoms per molecule in the form of selenocysteine [30]. The intake of selenium during the first month is expected to be supported by food based on a healthy eating style. However, the intake of selenium depends on the concentration of selenium in the soil or the biodiversity of the region. Fortified foods or functional foods can potentially supply the required essential micronutrients based on individual needs [31]. A Mediterranean diet model is widely recommended for the prevention of chronic diseases. This can be explained by the increased intake of fish, which is an important source of selenium. Additionally, oilseeds such as pecans are also rich in selenium [32].

The aim of this paper is to investigate the effectiveness of incorporating selenium in the management of OS, while considering a balanced diet based on a healthy lifestyle and dietary therapy. Selenium holds significant importance in regulating the thyroid hormones, reducing the proinflammatory processes in the body, and combating OS, especially when combined with the intake of flavonoids and polyphenols, which act as antioxidant sources. By implementing a balanced diet therapy and supplementing the diet with selenium, it is possible to achieve a reduction in adipose tissue and glycosylated hemoglobin levels, thereby contributing to the reduction of OS in the body.

## 2. Materials and Methods

The study was conducted in accordance with the Declaration of Helsinki and approved by the Institutional Review Board (or Ethics Committee) of the University of Oradea (protocol code CEFMF/1 from 31 January 2023 and date of approval). Each patient signed a personal data processing agreement.

In a 12-month prospective study, a total of 2144 patients were assessed. From this group, 206 patients were selected and randomized [33] from those who sought guidance from a nutritionist at the “TELIOS CARE” private medical office in Oradea, Romania. Patients were enrolled in the study upon presenting at the nutrition office. Each patient received a personalized diet based on their caloric requirement, determined using the Harris–Benedict formula. The diet was designed based on the principles of the Mediterranean diet, which involved reducing or eliminating foods high in salt, fat, and sugar, while promoting the consumption of vegetables, fruits, and fish instead of fatty meat.

The criterion for inclusion in the study was the patient's consent to participate and willingness to follow the study criteria. Exclusion criteria included a history of malignant tumors, organ failure, limitations for evaluations due to specific pathologies, and refusal to participate in the study.

**Figure 2:**
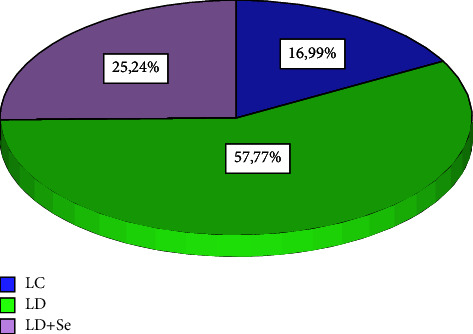
The percentage distribution of the research groups.

The cohort was divided into three groups based on the dietary therapy implemented, including the supplementation of selenium. The control group (LC) consisted of 35 individuals (17.0%) who did not follow any dietary treatment or received selenium supplementation. The diet therapy group (LD) comprised 119 individuals (57.8%) who underwent personalized dietary therapy as described without selenium supplementation. Lastly, the group receiving diet therapy along with selenium supplementation consisted of 52 individuals (25.2%), denoted as LD + Se (Figure 2). Patients in LD + Se group received a standardized selenium supplement of 200 mcg, sourced from the EU, for a duration of 30 days.

### 2.1. Body Analysis

The clinical evaluation was conducted using a body bioelectrical impedance analyzer (INBODY 370, USA), and the results were evaluated with the assistance of medical software. Bioelectrical impedance analysis (BIA) body analyzers are devices approved by the World Public Health Nutrition Association (WPHNA) that provide highly accurate measurements of body composition. The margin of error for these measurements is 0.1 kg. We monitored variations in the four independent groups based on factors such as sex, age, rural/urban environment, and clinical parameters including BMI, weight status, fat mass, visceral fat, and sarcopenic index. Body weight, as well as the other parameters, was determined in the morning, after daily toileting, without clothes. For women, monthly measurements during the menstrual cycle were avoided.

### 2.2. Paraclinical Analysis

Paraclinical evaluations were performed to support the diagnosis. In order to confirm metabolic disorders, paraclinical analyses were conducted, focusing on the HOMA index, cholesterol, triglycerides, C-reactive protein, and HGS. These paraclinical analyses were conducted in the analysis laboratory using enzymatic, colorimetric, and spectrophotometric methods, as well as immunoenzymatic tests. The homeostatic model of insulin resistance (HOMA-IR) was calculated using fasting glucose and insulin levels, according to the following formula: (glucose mg/dl × insulin (IU/L))/405.

### 2.3. Oxidative Stress Analysis

Oxidative balance was assessed through colorimetric analysis of oxygen free radicals (FORT) and oxygen free radical defense (FORD) in fingertip capillary blood. The FORT test (FORM CR 2000, Callegari, Parma, Italy) is based on the ability of transition metals, such as iron, to catalyze the decomposition of hydroperoxides (ROOH) into radicals via the Fenton reaction. These ROS react with an additive (phenylenediamine derivative, 2CrNH®2) to form a stable and colored radical cation, which can be detected at 505 nm using a spectrophotometer. The intensity of the color directly correlates with the amount of radical compounds and, consequently, with the oxidative state of the sample, in accordance with the Beer–Lambert law. The FORT reflects the levels of ROS in the blood. The results are expressed in FORT units, where 1 FORT unit corresponds to 0.26 mg/l of H_2_O_2_, and in normal individuals should have a value ≤ 2.3 mmol/L H_2_O_2_. Elevated FORT levels can induce oxidative stress, resulting in cellular harm such as lipid, protein, and DNA oxidation. The intra-assay and interassay coefficients of variation for this method were 3.7% and 6.2%, respectively.

The FORD test provides a measure of the plasma antioxidant system by using preformed, stable, and colored radicals, and typically yields a result within the 1.07–1.53 mmol/L Trolox range for healthy individuals. Below this value, the organism is exposed to oxidative stress. It involves determining the decrease in absorption, which is proportional to the concentration of antioxidants in the blood, based on the Beer–Lambert law. In an acidic environment (pH = 5.2) and in the presence of a suitable oxidant (FeCl_3_), the chromogen containing 4-amino-N, N-diethylalline sulfate forms a stable and colored radical cation, which can be photometrically detected at 505 nm. Antioxidant compounds present in the sample reduce the radical cation of the chromogen, leading to a decrease in color and discoloration of the solution proportional to their concentration. The absorbance values obtained for the samples are compared to a standard curve obtained using Trolox (6-hydroxy-2,5,7,8-tetramethylcroan-2-carboxylic acid), a cell-permeable derivative of vitamin E commonly used as an antioxidant. The intra-assay and interassay coefficients of variation for this test were 4.2% and 6.6%, respectively.

The MIXT test refers to the low FORT test and the increased FORT test in that person.

There is no consensus regarding the recommended dose and duration of antioxidant treatment. The UK Nutrient Reference Intake suggests a daily intake of 75 *µ*g for men and 60 *µ*g for women to optimize plasma GPX activity, which occurs at a selenium concentration of approximately 95 *µ*g/L. In the current study, each participant in the LD + Se group received a 200 mcg selenium supplement, a standardized product of EU origin, for a period of 30 days.

### 2.4. Sample Size Calculation

To detect a “large effect” (as defined by a coefficient of 0.9 [34]) in any between-group comparison for the measured parameters, a minimum sample size of 35 subjects per group was required. This calculation was based on a predetermined alpha level of 0.12 and a desired statistical power of 0.80.

### 2.5. Statistical Analyses

In the study, statistical analyses were conducted to investigate changes in biomarkers over time. Numerical summaries, such as mean ± standard deviation, were provided for each biomarker, along with case profile plots. Graphs were generated to visualize the relative changes from baseline (%) for the biomarkers. No evidence was found to suggest deviations from normality in the distribution of each biomarker at each time point.

To model the changes over time, a linear mixed model was employed. This model included a random effect for each individual to account for individual variability and an unstructured correlation to accommodate repeated measures of the same individual over time. The time of testing was treated as a fixed effect, initially as a categorical variable to compare the mean change at each time point and later as a continuous variable to compare the slopes of the biomarkers over time. Pearson correlations were used to examine the relationships between the biomarkers.

All statistical analyses were performed using Statistical Package for the Social Sciences (SPSS) software (New York, NY, USA, version 20.0). The significance level was set at *P* < 0.05. Residual plots from the fitted model were examined to assess the model's assumptions and the goodness of fit for each biomarker at each time point. Various statistical methods were employed, including ANOVA, post hoc analysis, chi-square test, and inferential statistics such as the Student's *t*-test. The Bonferroni test was utilized to compare the three research groups, while correlations between parameters were examined using the Bravais–Pearson tests and paired sample correlation.

## 3. Results and Discussion

A total of 206 individuals participated in the study, comprising 92 men (44.7%) and 114 women (55.3%), with an average age of 42.59 ± 11.10 years. Among them, 164 were from an urban environment (79.6%), while only 42 were from rural areas (20.4%). The average age of men in the study was 41.66 ± 9.40 years, which was lower than that of women, who had an average age of 43.33 ± 12.29 years. Individuals from urban areas had an average age of 42.84 ± 11.18 years, whereas those from rural areas had an average age of 41.62 ± 10.86 years, with no statistically significant differences.

Among the 35 individuals in the LC group, their BMI ranged from 30.92 ± 6.28, with values between 22.6 and 43.3 kg/m^2^. These values correspond to the first-degree obesity category, presented in Table 1. The sarcopenic index in this group was recorded at 8.69 ± 1.56 kg/m^2^. Only 14 individuals had controlled cardiovascular diseases. The average fat mass was 31.87%, and the visceral fat level was 8.74 ± 5.78.

In the LD group, which consisted of 119 individuals, the BMI ranged from 31.51 ± 6.71, with values between a minimum of 16 and a maximum of 52.59 kg/m^2^. These values also fall into the first-degree obesity category. The sarcopenic index in this group was recorded at 8.20 ± 1.90 kg/m^2^. Among all individuals, only 31.1% had controlled cardiovascular disease. The average fat mass was 33.78%, and the visceral fat level was 9.32 ± 6.38.

In the LD + Se group, which included 52 individuals, the BMI ranged from 31.23 ± 9.32, with values between 19.6 and 62.64 kg/m^2^. These values also correspond to the first-degree obesity category. The sarcopenic index in this group was recorded at 7.94 ± 1.72 kg/m^2^. Among them, 16 individuals had controlled cardiovascular disease. The average fat mass was 25.81%, and the visceral fat level was 5.34 ± 3.97. Statistically significant differences were observed between the groups in case of fat mass and visceral fat (*P* < 0.05).

The initial statistical test by ANOVA showed no significant differences in BMI (*F* = 0.092 and *P*=0.912) and obesity (*F* = 0.683 and *P*=0.506), sarcopenia index (*F* = 1.798 and *P*=0.168), and cardiovascular disease (*F* = 2.016 and *P*=0.136). However, significant differences were found in fat mass (*F* = 18.692 and *P*=0.001) and visceral fat (*F* = 8.786 and *P*=0.001). Furthermore, using the paired sample correlation, there were statistically significant differences (*P* < 0.01) in the baseline and final outcomes of BMI, obesity, sarcopenic index, cardiovascular disease, fat mass, and visceral fat.

**Figure 3:**
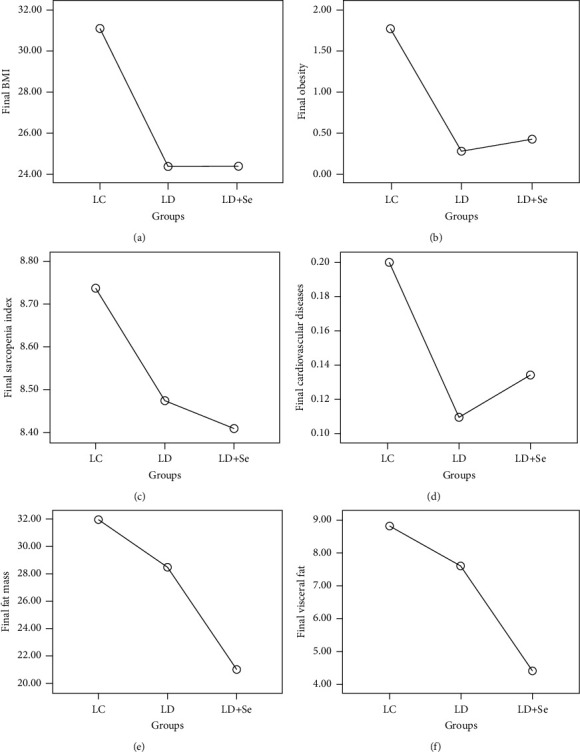
The graphical representation of the final results of BMI (a), obesity (b), sarcopenic index (c), cardiovascular disease (d), fat mass (e), and visceral fat (f) based on the three study groups.

The final data analysis of clinical parameters, depicting statistically significant differences between the groups, is presented in Figure 3(a). The poststudy BMI results were analyzed using the ANOVA statistical test for the three independent groups, yielding an *F* value of 43.592 and *P* value of 0.001. The post hoc Bonferroni test revealed significant differences between the LC and LD + S groups (*P* < 0.05) and the LC and LD groups (*P* < 0.05), indicating a significant decrease. However, no statistically significant difference was observed between the LD and LD + Se groups (*P* > 0.05).

Similarly, the ANOVA statistical test was employed to evaluate the effect of intervention on weight status, yielding an *F* value of 43.511 and *P* value of 0.001. The post hoc Bonferroni test showed significant differences between the LC and LD + Se groups (*P* < 0.05) and the LC and LD groups (*P*  <  0.05), indicating a significant decrease. However, no statistically significant difference was observed between the LD and LD + Se groups (*P* > 0.05), although there was an insignificant increase.

Regarding the sarcopenic index, the ANOVA statistical test yielded an *F* value of 0.523 and a *P* value of 0.593. This indicates that there were no significant differences in the sarcopenic index among the three groups poststudy. The post hoc Bonferroni test showed no significant differences between the LC and LD + S groups (*P* > 0.05) and between the LC and LD groups (*P* > 0.05). Similarly, there was no statistically significant difference between the LD and LD + Se groups (*P* > 0.05), indicating a lack of significance despite a decrease in values. The largest decrease in the sarcopenic index was observed in the LD + Se group but this decrease did not reach statistical significance.

To examine the effect of the intervention on cardiovascular diseases, an ANOVA statistical test was conducted for the three independent groups, resulting in an F value of 0.977 and a *P* value of 0.378. This suggests that poststudy (after one year), there were no significant differences in the occurrence of cardiovascular diseases among the three groups, as depicted in Figure 3(d). The post hoc Bonferroni test revealed no significant differences between the LC and LD + S groups (*P* > 0.05), between the LC and LD groups (*P* > 0.05), and between the LD and LD + Se groups (*P* > 0.05).

Furthermore, the effect of the intervention type on fat mass was assessed using an ANOVA statistical test for the three independent groups, yielding an F value of 36.256 and a *P* value of 0.001. Consequently, poststudy (after one year), significant differences in fat mass were observed among the three groups, as illustrated in Figure 3(e). The post hoc Bonferroni test demonstrated significant differences between the LC and LD + S groups (*P*=0.001); between the LC and LD groups (*P*=0.018), indicating a significant decrease; and between the LD and LD + Se groups (*P*=0.001), suggesting a statistically significant difference. Notably, the largest decrease in fat mass was observed in the LD + Se group.

Additionally, the impact of the intervention on visceral fat was analyzed using an ANOVA statistical test for the three independent groups, resulting in an F value of 10.233 and a *P* value of 0.001. Consequently, poststudy (after 1 year), significant differences in visceral fat were observed among the three groups, as depicted in Figure 3(f). The post hoc Bonferroni test revealed significant differences between the LC and LD + S groups (*P*  <  0.05), indicating a significant decrease, while no significant difference was found between the LC and LD groups (*P*=0.690), suggesting a nonsignificant decrease. However, a statistically significant difference was observed between the LD and LD + Se groups (*P*=0.001).

**Figure 4:**
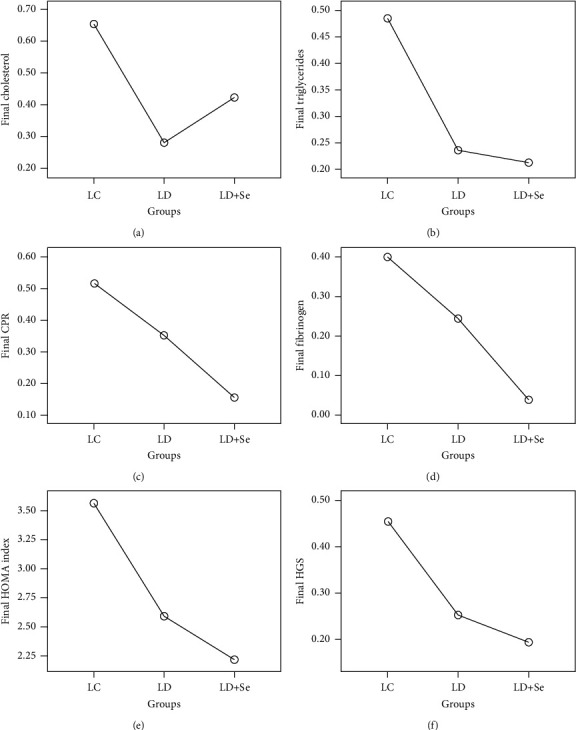
Graphical representation of the final results of cholesterol (a), triglycerides (b), CPR (c), fibrinogen (d), HOMA index (e), and HGS (f) according to the three intervention groups.

Following the paraclinical analysis on cholesterol evolution, an ANOVA statistical test was conducted for three independent groups, resulting in *F* = 9.228 and *P*=0.001. Therefore, poststudy (after one year), BMI showed significant differences among the three groups, as illustrated in Figure 4(a). Post hoc Bonferroni test revealed the following significant differences between the following groups: between LC and LD + Se (*P*=0.068), which was statistically insignificant; between LC and LD (*P*=0.001), indicating a significant decrease; and between LD and LD + Se (*P*=0.187), demonstrating a statistically insignificant difference.

For the analysis of triglyceride levels, the ANOVA statistical test was applied to the three independent groups, resulting in *F* = 5.095 and *P*=0.007. Consequently, the triglyceride levels showed significant differences among the three groups poststudy (after 1 year), as depicted in Figure 4(b). Post hoc Bonferroni test indicated significant differences between LC and LD + Se (*P*=0.010); between LC and LD (*P*=0.014), demonstrating a significant decrease; and between LD and LD + Se (*P* > 0.05), revealing a statistically insignificant difference.

To examine the effect of the intervention type on CPR, the ANOVA statistical test was employed for the three independent groups, yielding *F* = 6.812 and *P*=0.001. As a result, CPR exhibited significant differences among the three groups poststudy (after 1 year), as shown in Figure 4(c). Post hoc Bonferroni test revealed significant differences between LC and LD + Se (*P*=0.001), indicating a significant difference; between LC and LD (*P*=0.206), demonstrating a nonsignificant decrease; and between LD and LD + Se (*P*=0.029), revealing a statistically significant difference.

For the analysis of fibrinogen, the ANOVA statistical test was conducted for the three independent groups, yielding *F* = 9.170 and *P*=0.001. Consequently, fibrinogen displayed significant differences among the three groups poststudy (after 1 year), as depicted in Figure 4(d). Post hoc Bonferroni test indicated significant differences between LC and LD + Se (*P* < 0.05); between LC and LD (*P*=0.128), indicating a nonsignificant decrease; and between LD and LD + Se (*P*=0.007), demonstrating a statistically significant difference.

At the end of the study period, the HOMA index was assessed using the ANOVA statistical test for the three independent groups, resulting in *F* = 8.328 and *P*=0.001. Consequently, the HOMA index exhibited significant differences among the three groups poststudy (after 1 year), as shown in Figure 4(e). Post hoc Bonferroni test revealed significant differences between LC and LD + S (*P* < 0.05); between LC and LD (*P* < 0.05), indicating a significant decrease; and between LD and LD + Se (*P* > 0.05), indicating a statistically insignificant difference.

To examine the effect of the intervention type on HGS, the ANOVA statistical test was employed for the three independent groups, resulting in *F* = 3.195 and *P* = 0.043. As a result, HGS displayed significant differences among the three groups poststudy (after 1 year), as depicted in Figure 4(f). Post hoc Bonferroni tests revealed significant differences between LC and LD + Se (*P* < 0.05); significant differences between LC and LD (*P* > 0.05), indicating a nonsignificant decrease.

### 3.1. Correlations Related to the Evolution of Research Parameters and Oxidative Stress Parameters

Based on the Pearson correlation analysis between BMI and OS parameters, a significant positive correlation was observed with the FORT test. This indicates that as BMI increases, the value of the FORT test also increases, suggesting an increase in OS.

No significant correlation was found between the sarcopenic index and the evolution of OS, as indicated by the *P* values being greater than 0.05.

There is a direct correlation between cardiovascular disease and OS, which is linked to the high risk of developing atherosclerosis [35]. This connection was also observed in the current study, particularly between the FORT test and the MIXED test.

A statistically significant correlation was identified in relation to fat mass. This positive correlation indicates that an increase in fat mass is associated with a higher number of patients having a low FORT test, thus indicating a negative impact on OS.

Elevated levels of fibrinogen and the HOMA index are associated with an increase in patients having a high FORT test, suggesting their involvement in the development or worsening of OS. This can be attributed to the promotion of the proinflammatory process, both due to increased fibrinogen levels and the presence of MS.  Table 2 presents the Pearson correlation coefficients between the OS parameters and the research parameters.

Graphical representations of the Pearson correlation, using the dot and boxplot technique, depict the significant relationships between the following variables:

- (A) The difference in the FORD test and fat mass.
- (B) The FORT test and BMI.
- (C) The FORT test and obesity.
- (D) The FORT test and cardiovascular disease.
- (E) The FORT test and the HOMA index.

**Figure 5:**
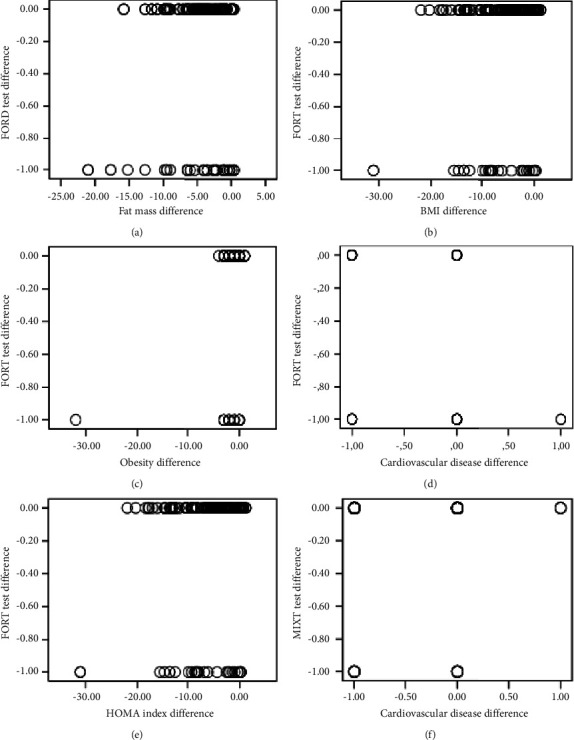
Graphical representation of the Pearson correlation with statistical significance, using the dot and boxplot technique, depicting the relationships between the following variables: (a) differences in the FORD test and fat mass, (b) the FORT test and BMI, (c) the FORT test and obesity, (d) the FORT test and cardiovascular diseases, (e) the FORT test and the HOMA index, and (f) the relationship between the MIXT test and cardiovascular diseases.

Furthermore, Figure 5 also includes the relationship between the MIXT test and cardiovascular diseases.

## 4. Discussion

OS is widely recognized as a significant mechanism that contributes to the development and progression of numerous pathological conditions. Many human diseases have been strongly associated with OS and its detrimental effects on various cellular processes [36–42]. Free radical molecules have been found to regulate a number of cellular functions and can also serve as intracellular and intercellular signals [43]. The redox state of proteins is also involved in regulating various cellular activities, such as cell differentiation and the activation of specific metabolic pathways [44]. OS has been associated with all individual components of MS and the development of cardiovascular complications in affected subjects [45]. The role of OS in the pathophysiological interactions among the contributing factors of MS has been recognized [46].

Poor skeletal muscle strength is independently associated with low plasma selenium, which was observed in 2007 by Lauretani et al. [47]. Minerals could play a role in both the prevention and treatment of sarcopenia, which is characterized by the age-related decline in muscle mass, muscle strength, and physical performance. [48]. In our study, the LD + Se group showed a significant improvement in the sarcopenia index compared to the other groups. It emphasizes the effect of selenium on muscle mass.

We present new findings on biomarkers of oxidative and nutritional stress, specifically FORT FORD and MIXT biomarkers. In 2016, Lewis concluded that the FORD and FORT tests could be utilized in sports and clinical settings, as well as in the field (such as training camps), for evaluating oxidative stress (OS) [49]. The repeatability of FORT and FORD assays is comparable to established laboratory measures of OS, demonstrating sufficient analytical precision for clinical applications. Moreover, the FORT assay outperforms other OS measurements reported in the literature [49]. The index of individuality suggests that reference intervals have limited utility in assessing meaningful changes in serial results within the individuals. Participants with the highest increase in FORT exhibited greater amounts of fat mass, visceral fat, and higher levels of the HOMA index.

The observed increase in FORT over the 10-hour period could be attributed to the enhanced release of circulating free fatty acids during fasting. A positive correlation between FORT and *α*-tocopherol indicates the increased mobilization of *α*-tocopherol from adipose tissue in response to fasting, which aligns with findings from previous studies [49, 50]. The potential theoretical mechanisms for the observed FORT increases include heightened fatty acid oxidation and increased production of H_2_O_2_. Previous studies have suggested that OS may play a role in various skin conditions, such as chronic ulcers, allergic reactions, and vitiligo [51–53].

The inverse relationship between FORT and RBC GSH supports these mechanisms [54].

During the formation of ROS, oxygen molecules acquire an unpaired electron, leading to the generation of a free radical. This free radical possesses the ability to generate additional ROS, including peroxides, which can cause oxidative damage. Such damage may involve processes such as lipid peroxidation and the release of inflammatory cytokines [55, 56].

ROS released from the follicular walls can contribute to skin damage, and it is believed that this process may play a role in the progression of inflammation in the pathogenesis of certain diseases. Based on this hypothesis, some of the drugs used for the treatment of the conditions such as acne work by reducing ROS levels [57]. Recent studies have placed emphasis on exploring the connection between OS and various skin diseases [58].

The ROS family encompasses both free radicals, such as the nitric oxide radical (NO), superoxide ion radical (O_2_−), and hydroxyl radical (OH), as well as nonradicals such as hydrogen peroxide (H_2_O_2_) and ozone (O_3_). These reactive species have been implicated in various biological processes, including mutations, carcinogenesis, inflammation, and aging [59, 60]. It is believed that an imbalance in the production of oxygen-derived pro-oxidants, also known as ROS, and the cellular antioxidant defense capacity can lead to the occurrence of OS. This imbalance is thought to increase the effects of ROS on cellular components, contributing to cellular damage and various pathological conditions associated with OS [61]. In addition to the toxic characteristics of ROS, the accumulation of ROS, such as hydrogen peroxide (H_2_O_2_) produced by neutrophils, is believed to have other adverse effects, including inflammation and tissue damage [62]. Furthermore, the interactions between ROS and lipids results in the peroxidation of polyunsaturated fatty acids [63]. The initial tests for detecting OS relied on the spectrophotometric technique [64].

Previous studies have reported that components of OS, such as ROS and lipid peroxide (LPO) [65], they may be involved in the parts of the body's pathogenesis, not just aging [66] but also the secretion of inflammatory cytokines [55, 56]. Since all free radicals generated by enzymes possess biological activity, an elevated enzymatic oxidation activity can result in the accumulation of ROS [67].

The antidiabetic effect of selenium was observed in a study conducted in rats in 2022 [68]. In this study, the patients in the LD + Se group experienced a significant decrease in HGS compared to the other groups.

Selenium (Se) is a vital nutritional trace element in animal production, serving as a structural component in at least 25 selenoproteins. It is involved in thyroid hormone synthesis and plays a crucial role in the antioxidant defense system. Supplementation of Se in the diet has been proven to support gastrointestinal health [69], enhance production performance [70], and improve reproductive physiology, particularly in stressful conditions. The mechanisms underlying these benefits include the regulation of nutrient digestibility by gastrointestinal microorganisms, maintenance of antioxidant status, and enhancement of immunocompetence [50, 71–73]. In our study, we emphasized the significance of selenium supplementation in patients with risk factors, particularly those with cumulative risk factors, high fat mass, or elevated glycemic levels.

OS plays a critical role in the development of comorbidities associated with obesity. Recent literature has provided evidence of obesity-induced OS in humans [74]. In our study, we observed a direct association between obesity and OS.

The limitations of this study may include the absence of blood analyses for serum levels of selenium and vitamin D. However, a notable strength is the utilization of a rapid test with high accuracy across various domains (such as athletes or individuals with cumulative cardiometabolic risk). This test does not entail substantial costs, yet its utility could yield significant benefits.

## 5. Conclusions

Selenium intake resulted in significantly better BMI evolution compared to the group receiving only diet therapy. The LD + Se group showed a significant improvement in the sarcopenia index compared to the other groups. Both diet therapy groups showed a significant decrease in fat mass and visceral fat compared to the control group but the LD + Se group exhibited significantly better results. The LD + Se group demonstrated the most significant decrease in CPR, an inflammatory marker, indicating a direct association and highlighting the beneficial effect of selenium supplementation in inflammatory processes. Fibrinogen levels significantly decreased in the LD + Se group. Patients in the LD + Se group experienced a significant decrease in HGS compared to the other groups. Selenium supplementation did not significantly affect cholesterol levels in the LD + Se group but it demonstrated that selenium alone is sufficient to reduce cardiovascular risk. Diet therapy played a crucial role, and both research groups showed significantly better results compared to the control group.

## Figures and Tables

**Table 1:** The statistical description of the baseline data for the clinical parameters.

Parameters			Groups			*P*
			LC	LD	LD + Se	
			*n* (%)	*n* (%)	*n* (%)	
BMI mean/SD			30, 93 (6, 98)	31, 52 (6, 71)	31, 23 (9, 34)	0.912

Obesity	Underweight		0 (0, 0%)	4 (3, 4%)	0 (0, 0%)	0.506
	Normal		4 (11, 4%)	17 (14, 3%)	16 (30, 8%)	
	Overweight		18 (51, 4%)	20 (16, 8%)	13 (25, 0%)	
	Obesity degree 1		4 (11, 4%)	45 (37, 8%)	10 (19, 2%)	
	Obesity degree 1		2 (5, 7%)	24 (20, 2%)	3 (5, 8%)	
	Obesity degree 1		7 (20, 0%)	8 (6, 7%)	10 (19, 2%)	

Sarcopenia index mean/SD			8, 69 (1, 57)	8, 20 (1, 91)	7, 95 (1, 72)	0.168

Cardiovascular diseases		No	30 (85, 7%)	82 (68, 9%)	36 (69, 2%)	0.136
		Yes	5 (14, 3%)	37 (31, 1%)	16 (30, 8%)	

Fat mass mean/SD			31, 87 (8, 53)	33, 78 (6, 89)	25, 82 (9, 33)	0.001

Visceral fat			8, 74 (5, 78)	9, 33 (6, 39)	5, 35 (3, 97)	0.001

*n* = number of patients, SD = standard deviation, BMI = body mass index, and *P* = statistically significance.

**Table 2:** Pearson correlation coefficients of OS parameters and research parameters.

Parameters	LC			LD			LD + Se		
	FORD test	FORT test	MIXT test	FORD test	FORT test	MIXT test	FORD test	FORT test	MIXT test
*Pearson correlation*									
BMI	−0.024	0.222	−0.001	0.118	0.150	0.215^*∗*^	−0.058	0.096	−0.022
Obesity	0.042	−0.389^*∗*^	0.075	0.020	0.250^*∗∗*^	0.322^*∗∗*^	−0.004	0.284^*∗*^	0.117
Sarcopenia index	−0.032	−0.352^*∗*^	−0.039	−0.072	0.065	−0.004	0.286^*∗*^	0.252	0.069
Cardiovascular diseases	0.042	−0.389^*∗*^	0.075	0.178	−0.058	0.125	0.713^*∗∗*^	0.368^*∗∗*^	0.323^*∗*^
Fat mass	−0.020	−0.074	−0.175	0.082	0.030	0.157	0.333^*∗*^	0.231	0.477^*∗∗*^
Visceral fat	−0.697^*∗∗*^	−0.389^*∗*^	−0.804^*∗∗*^	0.167	0.222^*∗*^	0.263^*∗∗*^	0.497^*∗∗*^	0.386^*∗∗*^	0.396^*∗∗*^
Cholesterol	^b^	^b^	^b^	0.132	−0.044	0.113	0.225	0.120	0.237
Triglycerides	0.053	−0.258	0.094	−0.052	−0.040	0.114	0.033	0.000	−0.143
CPR	^b^	^b^	^b^	0.069	0.268^*∗∗*^	0.256^*∗∗*^	0.117	0.368^*∗∗*^	0.105
Fibrinogen	^b^	^b^	^b^	−0.240^*∗∗*^	−0.106	−0.058	−0.263	−0.284^*∗*^	−0.165
HOMA index	0.020	−0.386	−0.252	0.035	0.275^*∗∗*^	0.303^*∗∗*^	0.489^*∗∗*^	0.194	0.261
HGS	0.033	−0.165	−0.173	0.258^*∗∗*^	−0.041	0.159	−0.019	0.178	0.192

BMI = body mass index, CPR=C-reactive protein, HGS = glycosylated hemoglobin, ^*∗∗*^ = correlation is significant at 0.01 level, ^*∗*^ = correlation is significant at 0.05 level, and ^b^cannot be computed because at least one of the variables is constant.

## Data Availability

The data used in this study are available from the corresponding author upon request.
